# A Word to Book Agents

**Published:** 1881-08

**Authors:** 


### A Word About Book Agents.

It is a well known and generally admitted fact that in the history of civilization, while progress and improvement are the rule, yet occasionally mistakes are made and steps have to be retraced. It is a natural consequence upon our increasing attempts at new methods that now and then a change fails to be an improvement, but on the contrary soon proves to have been an error; and this notwithstanding the best intentions. We believe such a mistake to have been made by certain publishers of medical works in their employment of traveling agents whose business is to solicit subscriptions in person. Admitting for the present that their professed object is the only one—zeal in the cause of medical education—we shall consider the subject simply from the standpoint of the physician.

Book agents, like other people, admit of classification in the good and bad, or, as some might have it, into bad and worst. While we do not deny that now and then an agent presents himself who is satisfied to wait his turn, satisfied with a few minutes of a physician's busy time, and is willing to accept an adverse decision; still, we all know these to be rare exceptions. The rule is self-assertion, arrogance and persistence. In the morning, perhaps, possibly before you have arisen, he rings and awakes you from your much-needed repose; or later in the day, during the morning hours, the office filled with waiting patients, your time more than fully occupied, he takes his place in the row. If with unusual condescension he waits his turn, you have a comparatively mild case to deal with. His story—he has taken special pains to see you, as Dr. —— has informed him that you are one of the leading physicians, a prominent member of the profession, etc., who, unlike so many, is determined that money-getting shall not interfere with his duty to his books. He has no doubt from your reputation that you will be more than pleased with the opportunity which his firm with such great sacrifice to themselves now offer you. While you are looking at the book, the names of all the prominent physicians who have already subscribed or perhaps have had the book given them, are read to you, and you begin to think that the neglect to purchase the work would be equivalent to excommunication. You buy, and with Ziemsen's Cyclopædia or some similar work on your library shelves to gather dust from year to year, you feel that you have done what was necessary to maintain your standing in the profession.

If, however, you should happen to be one of the obstinate and self-opinionated few who prefer to buy only after deliberate consideration, our obliging agent promises to call again in a day or two, and sometimes, in consideration of this dreaded alternative, you conclude that with peace thrown in the volume is after all very cheap. There may be an occasional medical man who has sufficient knowledge of what he wants and does not want that he can refuse and continue to refuse. But what is life to such a man; what self-reproach does he not feel when he thinks of the feelings which, according to our agent, the whole medical world must have toward such an anomaly. You are exhorted by your sense of right and duty, by the respect you owe to the publishers, by your duty to yourself and your honored profession. But all in vain; you prefer to lose an hour or two of time, with prospects of many more in the future, simply to prove yourself a stubborn, unreasoning man, with no object higher than to make a trade of your profession, with money-making and keeping its end.

We fail to see in this condition of things any of the so-called advantages which are proclaimed. On the contrary, the annoyances are self-evident. It is not long that we have had this evil, and it is not too late to remedy it. Let the publishers advertise by pamphlet, as formerly, and have the books in stock at the larger book stores, where they can be seen at times of leisure and convenience. Let physicians refuse to submit to the loss of time and other impositions of book agents, and the good days of our fathers, when not only book agents but those of all other classes were unknown, will once more return to this oppressed generation.

